# Ten simple rules for partnering with K–12 teachers to support broader impact goals

**DOI:** 10.1371/journal.pcbi.1008225

**Published:** 2020-10-01

**Authors:** Alexa R. Warwick, Angela Kolonich, Kristin M. Bass, Louise S. Mead, Frieda Reichsman

**Affiliations:** 1 Department of Fisheries and Wildlife, Michigan State University, East Lansing, Michigan, United States of America; 2 BEACON Center for the Study of Evolution in Action, Michigan State University, East Lansing, Michigan, United States of America; 3 CREATE for STEM Institute, Michigan State University, East Lansing, Michigan, United States of America; 4 Rockman et al, San Francisco, California, United States of America; 5 Department of Integrative Biology, Michigan State University, East Lansing, Michigan, United States of America; 6 The Concord Consortium, Concord, Massachusetts, United States of America; Carnegie Mellon University, UNITED STATES

## Abstract

Contributing to broader impacts is an important aspect of scientific research. Engaging practicing K–12 teachers as part of a research project can be an effective approach for addressing broader impacts requirements of grants, while also advancing researcher and teacher professional growth. Our focus is on leveraging teachers’ professional expertise to develop science education materials grounded in emerging scientific research. In this paper, we describe ten simple rules for planning, implementing, and evaluating teacher engagement to support the broader impact goals of your research project. These collaborations can lead to the development of instructional materials or activities for students in the classroom or provide science research opportunities for teachers. We share our successes and lessons learned while collaborating with high school biology teachers to create technology-based, instructional materials developed from basic biological research. The rules we describe are applicable across teacher partnerships at any grade level in that they emphasize eliciting and respecting teachers’ professionalism and expertise.

## Introduction

Broader impacts have become an increasingly important aspect of scientific research [1,2]. In order to foster new generations of scientists, broaden the participation of communities underrepresented in STEM, and promote public engagement with science, it is vital for scientists to share their work with K–12 schoolchildren and their teachers [3]. Outreach efforts as brief as a one hour classroom visit from a scientist or as extensive as weekly and year-long classroom instruction have produced substantial gains in students’ science attitudes and knowledge while also improving scientists’ pedagogical and communication skills [e.g., 3–7]. In addition, initiatives to support researchers in engaging broadly with their communities and society have continued to grow, such as the Center for Advancing Research Impact in Society [2].

One common approach to expanding the broader impacts of scientists’ work is to disseminate information and educational programming to public audiences, including K–12 teachers and students. This paper focuses on projects that require or would benefit from the involvement of K–12 teachers. For example, scientists may have new content, processes, or data to share with K–12 students. They may have an ecological citizen science project and want to train schoolchildren to collect data from their community. They may have a data set to share with high school students or want to provide access to a remote lab, server, or other pieces of equipment where students can do their own independent research. To help teachers and students to access and successfully engage in these opportunities, scientists can support the development of instructional materials for use in classrooms.

**Fig 1:**
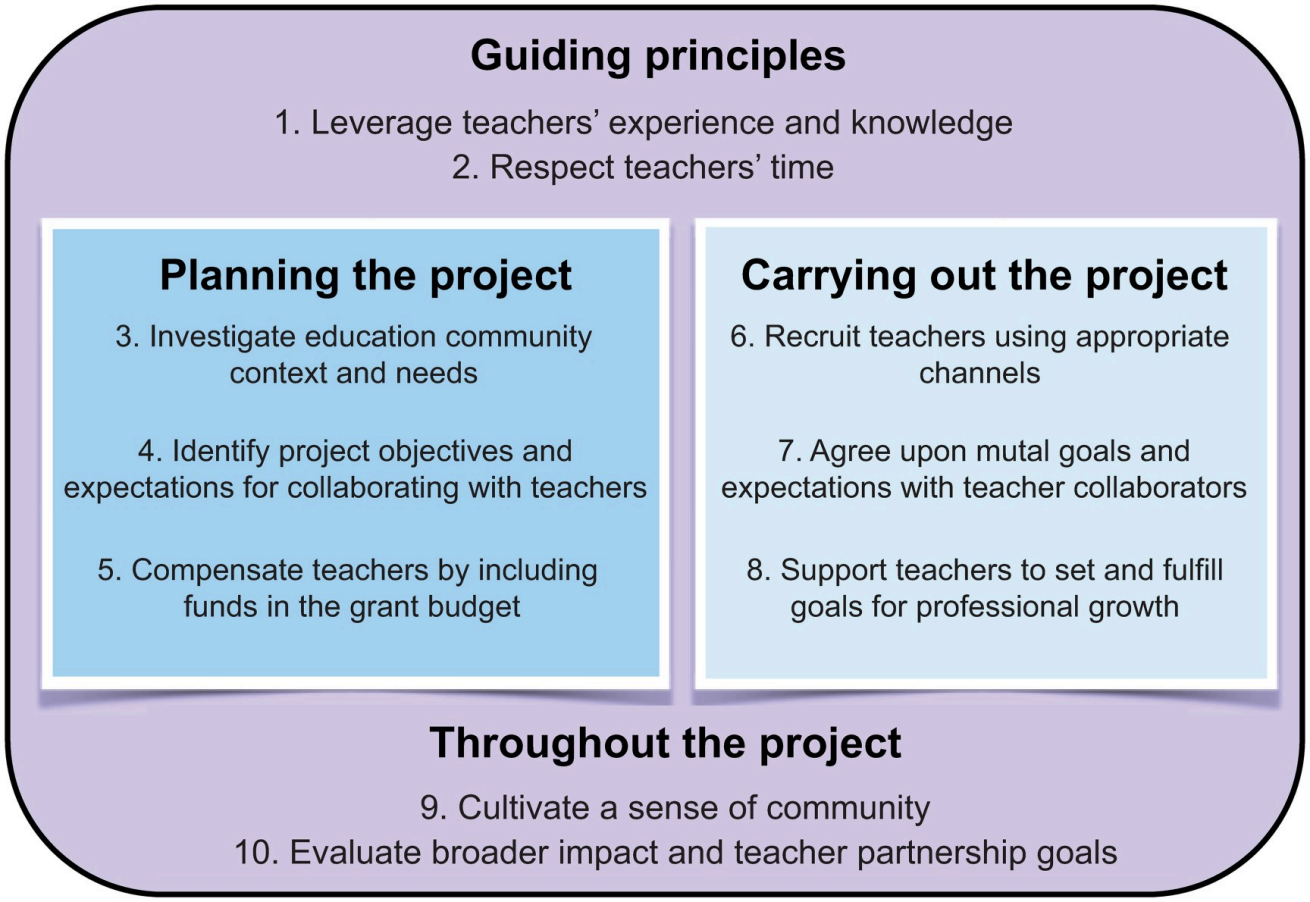
Summary and organization of the ten simple rules.

Our collective years of experience working with K–12 teachers indicate that there are many factors to consider when engaging K–12 teachers and their students. Researcher and teacher collaborations can greatly enrich both professions, with lasting mutualistic impacts on both research and practice [8]. Without appropriate planning and communication, however, researchers and teachers risk entering into partnerships with differing or conflicting expectations for the work involved, intended goals, or realistic project scope [3, 9]. These potential conflicts and barriers may cause concern, frustration, and stress for both partners, negatively impacting project outcomes [10]. Based on our experience, we have developed ten simple rules for structuring researcher and teacher partnerships to promote successful project outcomes and support lasting impacts on educational research and practice. We present these rules in four categories (Fig 1). We start with two guiding principles that underlie all aspects of collaborating with teachers in any grade. The next three rules pertain to planning researcher and teacher partnerships. The subsequent three rules address partnership implementation. The final two rules stress the importance of community-building and evaluation throughout the collaboration process.

The coauthors draw upon their expertise as scientists, education researchers, and former K–12 teachers, all of whom have collaborated on multiple science education projects for primary, middle, and high school grade levels. We simultaneously draw upon this previous experience and our recent experience working together on a project called Connected Biology (connectedbio.org/). Our goal on the Connected Biology project was to develop a quality set of instructional materials that involve high school students in the investigation of a biological phenomenon to support their understanding of genetics and evolution from the molecular level to the population level. The materials support students in exploring the case of light fur color evolution in deer mice, building upon college-level Evo-Ed materials [11]. The Connected Biology project was designed to engage teachers in both the development and implementation of the lessons by collecting student data to address education-focused research questions. This particular project was a much lengthier partnership because it included education research goals; however, the rules we describe below are still applicable across any teacher partnerships at any grade level in that they emphasize eliciting and respecting teachers’ professionalism and expertise.

## Guiding principles

### Rule 1: Leverage teachers’ experience and knowledge

Researchers have rich knowledge of scientific advancements that can have a positive impact in K–12 classrooms, especially when developed into curricular materials or activities for students. Partnering with practicing teachers during development and testing of materials is critical because they have valuable expertise of their student’s background and prior knowledge.

Practicing teachers can suggest the best strategies to support student learning and identify which activities might resonate with students [12]. Teachers know how to make science relevant to students from diverse backgrounds, which is critical given the increasingly diverse K–12 student population [13, 14]. They can help scientists identify questions that students might want to investigate, generate pertinent analogies or examples, and suggest meaningful applications of scientific ideas. This is especially important for reaching populations that have been traditionally underrepresented in science [15]. Including teachers from different demographic areas who have a range of student ages provides more feedback for revising materials. On the Connected Biology project, we recruited teachers in different states across the country who also taught a range of life science classes. Drawing from their rich, diverse experiences and learning what support different students required facilitated revision of our materials. In addition, teachers are intimately familiar with the needs for standards-aligned curriculum and are best situated to inform lesson development that meets these standards (most states have adopted some form of the Next Generation Science Standards, www.nextgenscience.org/). They are also aware of any policy impacts and limitations, including norms for the school context and culture.

### Rule 2: Respect teachers’ time

Keep in mind when working with teachers that they already have full-time jobs with a 100% teaching load, and some may run after-school programs, in addition to their personal commitments. When they agree to provide support for the project, respect their time and other responsibilities. Many think of teachers as having their “summers off,” but their summer availability may vary. Sometimes teachers have more availability after the school year ends, but other schools have year-round calendars with only short breaks. Look at their school or district calendar to identify times teachers may not be available (e.g., spring break and parent-teacher conferences). Additionally, teachers may start preparing for the next school year at the beginning of August or even earlier. As a result, it is especially important to be clear about work expectations and meeting times (Rules 4 and 7). Setting communication expectations early in the project is critical, especially in determining preferred communication modes and frequency.

Whether teachers take part in-person or remotely, it’s respectful to start and end meetings on time for teachers to attend to other personal and professional commitments. If the group doesn’t get through everything planned, the remaining topics might be addressed on email or during the next meeting. Teachers often say “okay” when asked about continuing after time ends because they want to be helpful. However, adhering to strict start and end times is a sign of respect. Another way of respecting their time is to make sure their talents are strategically used in the project (Rule 1). Teacher project tasks should be relevant to their expertise and professional goals. If someone else on the project could be completing the task (e.g., entering data), it may not be appropriate for the teacher(s).

## Planning the project

### Rule 3: Investigate education community context and needs

Just as in scientific research, obtaining sufficient background information to identify the needs of teachers and other school community stakeholders is the very first step. The research team may have ideas about the type of instructional materials needed in school contexts based on previous experiences, but avoid assuming that you know the most important focus for the particular teacher or school community with which you intend to work [9]. Classrooms are dynamic environments that exist within a school, community, and geographic location. Indeed, different states and districts adhere to curricula with different themes and learning outcomes. Many factors can support and constrain curricular development and implementation in classrooms, and these factors vary greatly across school settings. It is a good idea to talk directly with teachers or to consult with colleagues in a college of education or outreach specialists. Consider asking the following questions of teachers as part of the needs assessment:

- What grade level and content are teachers required to teach?
- How many students are in a typical classroom?
- What cultural and demographic factors may affect student learning?
- What resources (including technologies) are available in classrooms?
- What is the distribution of time teachers can commit to particular tasks such as implementing classroom instruction and completing assignments between project meetings?
- What are the current school or district initiatives, including curriculum standards?
- How can the research team support the teachers’ current work?

Conducting this needs assessment can help the research team determine whether the kinds of materials or outreach opportunities they want to develop are aligned with the school’s needs and context. For example, high school teachers may have one hour a day with their students. Elementary teachers may have longer periods of time during the day to engage their students in inquiry. Awareness of what teachers and students can realistically do can ensure that the initiatives developed will be implemented later. As a result of the needs assessment, the team may even decide to change the intended broader impact goals to better support the needs of the school, which in turn will also increase the potential positive impact from their efforts. In addition, understanding the school context can aid intentional recruitment of teachers for the project (see Rule 6). Taking time to learn and understand the unique context in which teachers work better supports the specific needs of those teachers and their students and the broader impacts the project aims to affect. For more example questions to ask, see [3].

### Rule 4: Identify project objectives and expectations for collaborating with teachers

Establishing project goals and objectives is one of the most fundamental steps for any successful project [16]. The research team should be clear about why it wants to include teachers in the project. Although it is possible for these goals to change over time, having initial goals and objectives is critical to establishing project expectations and desired outcomes. The team should also consider what it hopes to learn from interacting with teachers because the teachers likely have different perspectives and talents that complement the project team, which can result in mutual learning. Make sure the objectives are specific, measurable, achievable, relevant, and time oriented (SMART framework) [17]. Similarly, once teachers are involved, they should be setting their own objectives for professional growth (see Rule 8).

In the Connected Biology project, our two primary goals were to (1) design instructional materials for teachers and students to explore research-based biological phenomena and (2) conduct educational research to probe students on their ability to make connections across biological levels using these materials. Thus, the project objectives as related to engaging teachers included the development and piloting of instructional materials. When developing project expectations for teacher involvement (see Table 1), refer to these objectives. These expectations will likely be shaped by a variety of factors, including the team’s vision for the kind of materials to be developed and the breadth to which the materials will be used, the team’s professional time and resources, and the funder’s requirements [3, 9]. As with the objectives, make the expectations as specific as possible, keeping in mind a narrower project scope or target audience (only focusing on third graders at one school versus elementary students in an entire state) will make it easier to design educational materials. Be realistic about the time available and scale objectives and expectations accordingly (see example projects in [3]).

**Table 1:** Questions and examples to consider when developing expectations for teacher involvement.

	Question to answer	Examples
Time frame	What is the time frame and time of year in which teachers will be involved?	One day in July; January to June
Number and diversity of teachers	How many teachers are needed to complete the project? What types of diversity are represented (race and ethnicity, gender, career stage, etc.)?	One female teacher; Five teachers: two less than 5 years teaching and three more than 15 years
Geographic target	Is the team looking for teachers locally, statewide, nationally, or even internationally? If more than one teacher is needed, do they need to be in the same geographic area or in different areas?	One local teacher; five teachers each in different states
Grade level and disciplinary scope	What grade level is the team targeting?	Fourth grade; Ninth- or 10th-grade general biology
Deliverables	What is the expected work teachers will be doing? What products or outcomes are expected of them?	Two lesson plans; Survey after implementing a new lesson
Teacher hours	How many hours are teachers expected to be doing work on the project?	5 hours every week; 1 hour per month
Work format	Will teachers be able to work individually or is the work primarily collaborative? If it is collaborative, are teachers expected to work with the project team leads or with other teachers involved in the project?	50% individual and 50% collaborative work; 100% individual
Communication norms	What are the expected modes of communication (in-person, phone, email, etc.)? What frequency are updates expected, and to whom?	A weekly email with updates to the project team
Resources	What resources, if any, are needed that the team will provide to support teachers’ work?	Workspace; printed materials; campus parking; software; library access
Teacher growth	How will the project support teacher professional growth?	Visit a research lab; Present at a conference
Travel	Is any travel required for them to attend meetings, workshops, etc.? Will travel costs be reimbursed?	One in-person meeting per month
Compensation	How will teachers be paid for their involvement?	US$50 per hour; US$50 per lesson used

In conjunction with developing objectives, consider how the project team will know those objectives have been achieved. What will success look like? How will the team measure success? For example, if the team wants to learn from and with teachers, plan time in the project to reflect on what was learned. If the team wants to develop curriculum materials, collect evidence of the feasibility, appeal, and educational value of these materials. If the team plans to publish materials, including descriptions of outreach with teachers [e.g., 18], consider collecting systematic evaluation data. Rule 10 explores options for evaluation in more detail.

### Rule 5: Compensate teachers by including funds in the grant budget

It is important to pay teachers appropriately for their geographic region in monetary and/or continuing education credits for their time or deliverables. Including participant expenses in the research project budget will enhance the team’s ability to invest in teacher professional capacity (Rule 8) and, in turn, the success of the project’s broader impacts. In addition, including participant funds in a grant budget demonstrates a commitment to meeting broader impact goals by compensating teacher participation and providing a clear plan for their involvement. When deciding specific amounts to pay, check with the school or teachers about potential union rules impacting teachers’ contracted work hours. If the project requires teachers to leave school to be involved, consider paying for their substitute teacher. Be aware that at the end of the project in the dissemination phase, participant teachers may be doing professional development for other teachers, and this may require a reevaluation of compensation. Similarly, if teachers will attend conferences, include travel expenses. If students will be going on field trips, include bus expenses. Budgeting for these kinds of expenses could even boost grant funding success in comparison to grant applications that only mention outreach with K–12 schools but have no funds to support such efforts. Also, be familiar with particular institutional requirements for payment (filling out tax forms, travel authorization, etc.). There are other nonmonetary ways to support teachers as well, which will be discussed in Rule 8.

## Carrying out the project

### Rule 6: Recruit teachers using appropriate channels

After clarifying how teachers will be involved, recruit them using the appropriate channels at the district or school level. When possible, leverage existing relationships with teachers or schools through your institution(s). Reach out to the education faculty and/or the community engagement and outreach office (or the service-learning and civic engagement office, depending on your project) at your institution to get assistance. When looking for teachers in a particular location, reach out to the curriculum director for a school district to recruit. Participation in other forms of outreach, such as Skype a Scientist (www.skypeascientist.com/), may help establish a relationship with a teacher. Depending on the needs of the project (review Table 1) and geographic breadth of recruitment, another option is to use teacher networks including Advanced Placement (apcommunity.collegeboard.org/), state (e.g., Michigan Science Teacher Association: www.msta-mich.org/; California Science Teacher Association: cascience.org/; Massachusetts Association of Science Teachers: www.massscienceteach.org/), and national level teacher organizations (e.g., Computer Science Teacher Association: www.csteachers.org/;National Association of Biology Teachers: nabt.org/; National Science Teaching Association: www.nsta.org/).

Perhaps, the teachers the team wants to recruit are the same as those the team informally approached as part of the needs assessment (Rule 3). In that case, keep in mind that your institution may have an established protocol for formally reaching out to community partners. The schools and districts also may have established protocols for recruitment. And, even when recruiting teachers directly, they may still require administrator or district level approval. Similarly, if a project objective is to publish curricular materials, especially evaluation data from students or teachers, you may need to obtain institutional review board (IRB) (human subjects research) approval from your institution in advance. The school and/or district may also have specific IRB requirements. Allow for extra time to complete these steps prior to the start of any evaluation data collection.

Throughout recruitment, be attentive to diversity gaps in the current teacher workforce and the potential impacts on student outcomes [14, 19, 20]. Strive to intentionally broaden participation for both students and teachers in the project by identifying and eliminating barriers to recruitment. A better understanding of the education community context (Rule 3) should help the team improve equity [21]. Finally, if more than one teacher will be recruited, consider various types of diversity [22] among those in the group and work toward an inclusive community (Rule 9).

### Rule 7: Agree upon mutual goals and expectations with teacher collaborators

By the time the research team first meets with teachers, it will likely be eager to get straight to work. However, there is still a bit more norm-setting to do. Before the team and the teachers start to collaborate, describe the project’s broader impact goals. Ask teachers why they wanted to participate and what they hope to gain from the experience (Rule 8). It is also important to work toward mutually agreed upon expectations for what the team and teachers will be doing on the project and how everyone will work together to get it done. Revisit the questions considered during the recruitment process (see Table 1; Rule 4) to confirm the scope of work, timeline, deliverables, and compensation. These expectations can be modified at a later date, with mutual agreement from the teachers, but initial expectations provide teachers with a clear understanding of their role on the project. It is equally important that the research team meet their defined role and expectations as well. For example, if teachers are expecting lab materials or other resources to implement a lesson in their classroom, make sure to deliver materials on time.

In addition, addressing communication norms early on in the project is critical for success because scientist and teacher professional cultures differ [10]. For example, some teachers may not be as accustomed to using email for regular communication and may not be checking their email as frequently as the project team might expect. Make sure to have a discussion about these expectations with the teachers to determine what will work best for the team and project goals.

### Rule 8: Support teachers to set and fulfill goals for professional growth

Engaging teachers in a research project can present ample opportunities for teacher professional growth. Collaborations between researchers working on emergent topics in science and teachers who are implementing new curricular materials allows both parties to give and receive feedback and facilitate project and professional development. Early in the project, the team identified broader impacts goals and objectives (Rule 4). Similarly, ask teachers what they want to gain from this experience and have them set their own goals [23]. Example teacher goals may include learning about emerging scientific discoveries, collaborating and/or networking with peers, acquiring new materials to teach science concepts and practices, or developing their pedagogical practice. Supporting teachers in identifying professional goals and the types of project involvement they are interested in mutually supports teachers’ professional growth and project outcomes. When the expertise of teachers is leveraged in collaboration with researchers toward an educational goal, teacher agency is increased [24]. Fostering teacher agency to complete work that aligns with both project goals and teacher goals can enhance the work inputs and learning outputs for all team members.

Furthermore, investing in a teacher’s professional growth supports the forward momentum of the project. The more teachers learn about your research context, the more they can support your work. Some examples might require additional funding, such as including teachers in research or workshop presentations at conferences or taking teachers or students on field trips (see Rule 5). Other examples may not require additional monetary investment but, instead, leverage institutional privileges such as providing access to research articles or equipment, expanding the teachers’ professional network by engaging them with the team’s research colleagues, or connecting them with other opportunities on your campus or beyond. Teachers (and their students) should also receive attribution, acknowledgment, or authorship for their contributions to the project’s research (e.g., contributions of DNA sequencing data to a public database). The specific activities selected should be codetermined by the project team and the teacher to be aligned with the teacher’s professional goals. If teachers present at a conference, covering half of their expenses is appropriate, with the recommendation that the school pay the other half of travel. In addition to meeting teacher professional goals, having teachers present also increases visibility and broader impacts of the project. The message might be particularly effective because it is coming from a teacher participant.

## Throughout the project

### Rule 9: Cultivate a sense of community

Building strong relationships among the project team and participating teachers can support effective and frequent communication, and it makes the work enjoyable. Project participants are more likely to maintain active involvement in the work and communication with the team if they know one another personally and feel welcomed, respected, supported, and valued [21]. Getting to know teachers as people by asking them about their interests and life beyond work can support the development of a research-practice community. Community building doesn’t have to take much time, but investing in teachers and the project team as people should be a frequent focus of project work. Taking time at the beginning or end of meetings to talk about a recent vacation, family, or interests outside of work makes for a more congenial project work community. Similar to researchers, teachers in the classroom can sometimes feel isolated from their colleagues. Many teachers enjoy the opportunity to network and share with other practicing teachers.

By building relationships with the teachers, they may become involved in other research projects or experiences or become more comfortable asking questions about your scientific discipline. Teachers usually appreciate when you connect them with another appropriate professional to answer their question, if it is outside your area of expertise. The teachers may also find out about other professional development or educational programs from the other teachers on the project from different geographical areas and schools that may further their pedagogical practice.

### Rule 10: Evaluate broader impact and teacher partnership goals

Evaluation of the project throughout its life span is important to determine project success; did the research team meet the broader impact goals and objectives established (Rule 4)? During the project, periodic stakeholder check-ins are essential to maintain partner engagement and facilitate project progress. Flexibility is key. Researchers and teachers should be prepared to adjust goals or make midproject corrections as needed. Consider administering short, focused, anonymous surveys to gather feedback about teachers’ satisfaction with the project, attainment of goals, and preparedness to do what’s been asked of them. Surveys can be administered through various platforms. After teachers respond, share a summary of the feedback and the project changes made with the teachers to let them know they have been heard. If the research team is uncertain how best to address their feedback, even anonymous feedback, reach out to the entire group for further clarification and suggestions.

We also recommend assessing both broader impact and teacher professional goals at the end of a project to gauge accomplishments, improve future work, and inform subsequent grant proposals. The research team can use anonymous surveys, individual interviews, or a group debrief. An external, third-party evaluator can help collect independent evaluation data and work with the project team to interpret the findings [25, 26]. If the team wants to engage an evaluator, be sure to allocate funds in the project budget.

Finally, if a project objective is to study the effectiveness of instructional materials to document broader impact, the team can do so in a variety of ways. Options include observing the materials implemented in a classroom or conducting surveys and interviews with teachers and students. Consult literature on STEM outreach [e.g., 27] for examples of how others have gauged the feasibility and educational value of their work. As noted in Rule 6, make sure to obtain IRB approval and school permissions before collecting data from human subjects. Keep in mind that change takes time and some measures of success may arise that were unanticipated [7]. By investing in teachers as project partners, the research team can work toward sustained change rather than isolated outreach efforts [28].

## Conclusion

We anticipate our ten simple rules will support future collaborations with K–12 teachers, contribute to researcher and teacher professional growth, and increase broader impacts on research projects. Teachers are experts on what happens in their school communities. They have a wealth of pedagogical knowledge and experience that can enhance the work of scientists and are often best placed to reach communities traditionally underrepresented in science. Furthermore, if the team is interested in working directly with their students (e.g., visiting a classroom or organizing a field trip), teachers will be the best guide on how to structure these activities effectively. We also emphasize compensation considerations, such as budgeting for teacher pay and other resources (e.g., travel to conferences). It’s not as hard as you might think to invest in teachers’ professional growth. Sometimes it just requires considering the resources you access on a regular basis (e.g., equipment, software, libraries, and colleagues) and giving teachers an opportunity to do the same.

These rules were developed as general guidelines for structuring researcher and teacher partnerships. Our expectation is for them to be used while negotiating project-specific needs. We emphasize partnerships built on transparent communication and encourage intentionally setting up those communication expectations at the beginning because researchers and teachers may be familiar with very different work contexts [10]. For most partnerships with science researchers, we expect instructional material development to be a primary goal, although many of these rules are just as applicable for conducting science education research involving teachers. As in many fields, relationship building is key to establishing researcher and teacher partnerships. We encourage project partners to get to know each other as people and professionals with shared interests and goals.

Finally, expect that iteration across the rules will be necessary. Periodic check-ins about whether needs are being met and revising expectations along the way are important, just as in any collaborative project. In addition, spending time and/or funds on evaluation (i.e., collecting empirical evidence related to project objectives) can have great payoff for use in later grants and for job performance evaluation and promotion to demonstrate broader impacts. We hope you will find that these rules help to demystify the process of partnering with teachers and that your experiences with teachers will be as fulfilling, fun, and productive as ours.
